# Association between pre-and postnatal growth and longitudinal trends in serum uric acid levels and blood pressure in children aged 3 to 7 years

**DOI:** 10.1186/s12887-020-1922-8

**Published:** 2020-01-20

**Authors:** Bomi Park, Bohyun Park, Hye Ah Lee, Seonhwa Lee, Hyejin Han, Eunae Park, Su Jin Cho, Hae Soon Kim, Young Ju Kim, Eun-Hee Ha, Hyesook Park

**Affiliations:** 1 Department of Preventive Medicine, College of Medicine, Ewha Womans University, 25, Magokdong-ro 2-gil, Gangseo-gu, Seoul, 07804 Republic of Korea; 2 National Cancer Control Institute, National Cancer Center, Goyang, Republic of Korea; 3 Clinical Trial Center, Mokdong Hospital, Ewha Womans University, Seoul, Republic of Korea; 4 Department of Pediatrics, College of Medicine, Ewha Womans University, Seoul, Republic of Korea; 5 Department of Obstetrics and Gynecology, College of Medicine, Ewha Womans University, Seoul, Republic of Korea; 6 Department of Occupational and Environmental Medicine, College of Medicine, Ewha Womans University, Seoul, Republic of Korea

**Keywords:** Low birth weight, Catch-up growth, Uric acid, Blood pressure, Longitudinal cohort study

## Abstract

**Background:**

Uric acid has been identified as an important factor in the development of hypertension. If low birth weight (LBW) combined with catch-up growth (CUG) is associated with continuously elevated serum uric acid levels (SUA) level trajectories, LBW children who experience CUG may have an increased risk of hypertension later in life. Therefore, this cohort study analyzed longitudinal trends in SUA levels and changes in blood pressure in relation to pre- and postnatal growth over an extended follow-up period.

**Methods:**

This prospective cohort study of 364 children from the Ewha Birth and Growth Cohort assessed the effects of pre- and postnatal growth status on SUA at 3, 5, and 7 years of age using a linear mixed model and the change in blood pressure over the 7-year follow-up period using a generalized linear model (analysis of covariance). CUG was defined as a change in weight (between birth and age 3) with a *z*-score > 0.67 for LBW subjects. The multivariate model considered sex, gestational age, and uric acid, height, and weight at 3 years of age.

**Results:**

Children with LBW and CUG had higher SUA for the first 7 years of life compared to the normal birth weight group. This trend was particularly evident when comparing LBW children at term to children with normal birth weight. Within the group with LBW at term, children with greater CUG had higher SUA than children with normal birth weight, and this difference increased with age. Changes in the systolic blood pressure between 3 and 7 years of age were higher by 7.9 mmHg in children who experienced LBW and CUG compared with those who had a normal birth weight after adjusting for sex, gestational age, and height, weight, and uric acid at 3 years of age (*p*-value = 0.08).

**Conclusions:**

The uric acid levels and changes in systolic blood pressure were consistently higher among LBW children who experienced CUG compared with NBW children for the first 7 years of life. LBW children who experienced greater weight gain from birth to age 3 had even higher uric acid levels compared with NBW children.

## Background

Hypertension is a leading risk factor for cardiovascular disease, heart disease, and kidney disease and is a major underlying cause of morbidity and mortality [1–3]. Pediatric hypertension has become more prevalent [4, 5], and recent longitudinal studies have indicated that high blood pressure during childhood is associated with hypertension in adulthood [6, 7].

Experimental and clinical observation studies have proposed that uric acid, a product of purine catabolism, plays an important role in the pathogenesis of hypertension. For example, serum uric acid levels (SUA) were positively associated with the degree of hypertension in children and adolescents [8–11]. Additionally, controlling uric acid levels has been observed to lower blood pressure in adolescents with essential hypertension [12].

Low birth weight (LBW) is a well-established risk factor for the development of hypertension later in life [13–15]. Recent studies have also demonstrated that accelerated postnatal catch-up growth, a common compensatory mechanism for LBW, is another important factor in the development of cardiometabolic disease, including hypertension [16, 17]. Thus, LBW children who experience accelerated postnatal growth may have an increased risk of hypertension.

These separate pieces of evidence supporting an association of uric acid with hypertension and either LBW or catch-up growth with hypertension suggest that uric acid may have a pathogenic role in the development of hypertension later in life in LBW children. In light of this finding, it is important to explore the association between pre- and postnatal growth status and uric acid.

A study reported that preterm birth combined with accelerated postnatal growth is associated with higher SUA levels at age 3 [18]. Other studies have investigated the association between birth weight and SUA levels in children and adolescents, but they measured SUA levels at one time point only [13, 16, 19]. To our knowledge, no studies have prospectively investigated SUA level trajectories. We do not know how long birth weight and catch-up growth are associated with SUA levels or blood pressure. Therefore, in this cohort study, we analyzed longitudinal trends in both SUA levels and changes in blood pressure in relation to pre- and postnatal growth over an extended follow-up period. The STROBE guidelines were followed to report this study [20].

## Methods

### Study population

This cohort study was conducted with children from the Ewha Birth and Growth Cohort, a prospective birth cohort established at Mokdong Hospital, Ewha Womans University, Seoul, South Korea. The cohort includes children born to mothers who received regular prenatal care including targeted ultrasonography and a diabetes screening between 24 and 28 weeks of gestation from 2000 to 2005. The details of this cohort are reported in a previous study [17].

The first examination was performed when the children were 3 years old (between November 2005 and July 2010), with follow-up examinations being performed at 5 and 7 years of age. Singleton children with recorded birth weight and gestational age, and children with a weight measurement at 3 years of age and at least one measurement of uric acid during the follow-up period were included in our analysis. LBW children who did not exhibit catch-up growth were excluded from the analysis, as most LBW children (95%) experienced catch-up growth. A total of 365 children were included in the study.

The study protocol was approved by the Institutional Review Board on Human Subjects at Ewha Womans University (IRB number: EUMC 2015–04–048-017). Parents or guardians of all participants provided informed written consent.

### Data collection

Gestational age and birth weight of each subject were obtained from medical records. Preterm birth was defined as birth earlier than 37 gestational weeks. LBW was defined as a birth weight of less than 2.5 kg.

Anthropometric measurements were performed by well-trained researchers. Current height with no shoes was measured to the nearest 0.1 cm using a stadiometer; weight in light clothing was measured to the nearest 0.1 kg using a well-calibrated scale (DS-102, Dong Sahn Jenix Co. Ltd., Seoul, Korea). Body mass index (BMI; kg/m^2^) was calculated as weight divided by height squared. Blood pressure was measured twice using an automatic device (Dinamap Procure 200, GE, Milwaukee, WI) with an appropriate cuff size after the participants had been rested for five minutes. The average of the two measurements was used.

Birth weight and weight at age 3 were converted to an age- and gender- specific z-score using criteria from the 2007 Korean Children and Adolescents Growth Standards [21]. Catch-up growth was defined as a change in weight (between birth and age 3) by a z-score > 0.67 for LBW subjects [22].

Venous blood was drawn from subjects after an 8-h overnight fast to determine SUA level, which was used as the repeated outcome variable. SUA concentrations were measured using the uricase- and peroxidase-coupled reaction method (Hitachi Auto-analyser 7600, Fukuoka, Japan) in the Clinical Biochemistry Department of the Seegene Medical Foundation (Seoul, South Korea).

### Statistical analysis

Demographic and anthropometric characteristics of participants were summarized using descriptive statistical analyses. The means with the standard deviation or median and interquartile range were calculated for continuous variables after performing the normality test, and frequencies and percentages of totals were calculated for dichotomous variables.

A linear mixed-model analysis using SAS Proc Mixed was used to estimate the effects of birth weight and postnatal growth on repeated measures of SUA levels. A REPEATED statement was used to indicate within-subject correlation. Year of follow-up visit was included as a categorical variable. Group, time at which each measurement was taken, and the interaction between group and time were included in a linear mixed model.

The first model assessed whether there was a difference in uric acid level over time between normal-birth-weight (NBW) children and LBW children who experienced catch-up growth. LBW can be caused by either preterm birth or intrauterine growth restriction. Therefore, to assess the effects of LBW from intrauterine growth restriction only, we excluded children born with LBW due to preterm birth in the second model. In the third model, SUA levels were compared to the degree of catch-up growth among LBW-at-term children. The stratification cut point was defined as the median value of weight z-score changes between birth and age 3 among LBW-at-term children who experienced catch-up growth.

For all models, we utilized an unstructured covariance matrix to minimize Akaike’s Information criterion and model the variance and correlation among repeated measurements. The results are presented as least-squares means with 95% confidence interval. Post hoc analysis with Bonferroni correction was applied to adjust for multiple comparisons. The type III test of fixed effects was used to determine statistical significance. Sex, gestational age, and BMI were further assessed in a multivariable model.

The assumptions for the mixed model were checked and confirmed to be verified in all models: Normality was tested by a quantile-quantile plot and linearity was confirmed using plots of the residuals versus each of the variables. Figures have been added to show this in more detail (Additional files 1 and 2). In addition to ascertaining that the model is not influenced by the small set of observations, the change in coefficients was evaluated after dropping observations with high Cook’s distances. The coefficients changed by as much as 9~31% depending on the models, and there were no differences in the direction or significance of the results after dropping observations. Therefore, we decided to keep the observations to maintain the statistical power.

We also analyzed the effect of birth weight and catch-up growth on the changes in systolic blood pressure (SBP) between 3 and 7 years of age using generalized linear model. In the multivariable model, sex, gestational age, and uric acid, height, and weight at 3 years of age were considered.

A *p*-value < 0.05 based on a two-tailed test was considered to be statistically significant. All analyses were conducted using SAS, version 9.4 (SAS Institute Inc., Cary, North Carolina, USA).

## Results

Demographic characteristics and anthropometric parameters at birth and ages 3, 5, and 7 of eligible study subjects are presented in Table 1. At baseline, 175 (48%) of the total participants were male and 189 (52%) were female. The median birth weight and gestational age were 3.2 kg and 39.0 weeks, respectively. There were 308 (85%) NBW children, 20 (5%) LBW-at-term children who experienced catch-up growth, and 36 (10%) premature LBW children who experienced catch-up growth. The number children measured serum uric acid level at 3 years was 353 (NBW: 298, LBWCUG: 55), at 5 years was 118 (NBW: 105, LBWCUG: 13), and at 7 years was 132 (NBW: 121, LBWCUG: 11). The mean SUA concentrations at ages 3, 5, and 7 were 3.8, 4.0, and 3.9 mg/dL, respectively. Seventeen children had SUA concentrations greater than normal (2.0–5.5 mg/dL) at one, at least, of the follow-up points, and nine of 13 children with more than one measurement showed a consistently elevated uric acid level of greater than quartile three at each follow-up point. The number of children measured systolic blood pressure at 3 years was 364 (NBW: 308, LBWCUG: 56), at 5 years was 186 (NBW: 160, LBWCUG: 26), and at 7 years was 162 (NBW: 145, LBWCUG: 17). The mean SBP at age 3, 5, and 7 were 94.3, 100.4, and 101.6.

**Table 1:** Demographic and anthropometric characteristics

Characteristics	N ^e^, Mean ± SD, N ^e^, Median (IQR), or N (%)			
	All	NBW	LBWCUG	*P*-value^d^
Sex ^a^				
Male	175 (48)	155 (50)	34 (61)	0.16
Female	189 (52)	153 (50)	22 (39)	
Birth weight (kg)^b^	364, 3.2 (2.8–3.5)	308, 3.2 (3.0–3.5)	56, 2.2 (1.7–2.4)	< 0.0001
Gestational age (weeks) ^b^	364, 39.0 (37.6–40.0)	308, 39.2 (38.1–40.1)	56, 36.0 (33.5–37.1)	< 0.0001
Weight gain 0–3 years (kg)^c^	364, 11.7 ± 1.8	308,11.5 ± 1.7	56,12.3 ± 1.8	0.00
Weight at 3 years (kg) ^c^	364, 14.7 ± 1.8	308, 14.8 ± 1.8	56, 14.3 ± 1.8	0.06
Height at 3 years (cm) ^c^	364, 97.3 ± 4.4	308, 97.4 ± 4.4	56, 96.8 ± 4.3	0.37
BMI at 3 years (kg/m^2^) ^c^	364, 15.5 ± 1.3	308, 15.6 ± 1.3	56, 15.2 ± 1.3	0.06
BMI at 5 years (kg/m^2^) ^c^	187, 15.7 ± 1.6	165, 15.7 ± 1.6	22, 15.6 ± 2.0	0.88
BMI at 7 years (kg/m^2^) ^c^	157, 16.0 ± 2.0	142, 16.1 ± 2.1	15, 15.9 ± 2.3	0.78
Uric acid at 3 years (mg/dL) ^c^	353, 3.8 ± 0.8	298, 3.7 ± 0.8	55, 3.9 ± 0.9	0.20
Uric acid at 5 years (mg/dL) ^c^	118, 4.0 ± 0.8	105, 3.9 ± 0.7	13, 4.2 ± 0.9	0.20
Uric acid at 7 years (mg/dL) ^c^	132, 3.9 ± 0.9	121, 3.9 ± 0.9	11, 4.3 ± 0.9	0.17
SBP at 3 years (mmHg) ^c^	364, 94.3 ± 11.6	308, 94.6 ± 11.4	56, 92.9 ± 12.2	0.32
SBP at 5 years (mmHg) ^c^	186, 100.4 ± 8.4	160, 100.8 ± 8.5	26, 98.1 ± 7.0	0.13
SBP at 7 years (mmHg) ^c^	162, 101.6 ± 10.9	145, 101.6 ± 11.3	17, 101.4 ± 7.5	0.94

*SD* indicates standard deviation, *IQR* interquartile range, *NBW* normal birth weight, *LBW* low birth weight, *CUG* catch-up growth, *BMI* body mass index, and *SBP* systolic blood pressure

^a^Values are presented as N (%)

^b^Values are presented as Median (IQR)

^c^Values are presented as Mean ± SD

^d^*P*-value was calculated with t-test or chi-square test

^e^Instances of item non-response account for missing data

**Fig. 1:**
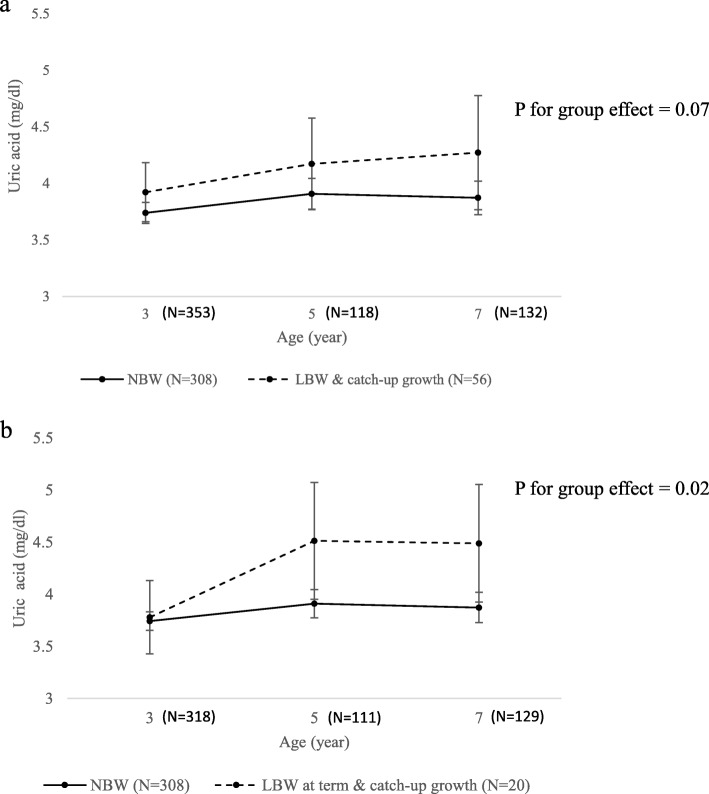
Serum uric acid trajectories from ages 3 to 7 by pre-and postnatal growth status. The points represent the estimated least-squares means, with vertical bars representing the 95% confidence intervals. The solid line indicates the trajectory of the normal birth weight (NBW) group (*N* = 308). The dashed line indicates the trajectory of the (**a**) low birth weight (LBW) and CUG (catch-up growth) group (*N* = 56), and (**b**) LBW at term and CUG group (*N* = 20). Results were adjusted for sex, gestational age, and body mass index at the age of uric acid measurement

The first model assessed whether SUA levels differed over time between NBW and LBW children who experienced catch-up growth. When modeled without adjustment, LBW children who experienced catch-up growth had consistently higher SUA levels between 3 and 7 years of age, and the effects of group and time were significant with *p*-values of 0.029 and 0.032, respectively. After adjusting for sex, gestational age, and BMI, LBW children who experienced catch-up growth had higher uric acid levels than NBW children over the entire follow-up period (Fig. 1-a), although the difference was not significant in the multivariable model.

To assess the effect of LBW due only to intrauterine growth restriction without the effect of preterm birth, children born as LBW due to preterm birth (7 children at 5-year-old follow-up and 3 children at 7-year-old follow-up) were excluded in the second analysis and only LBW-at term children were included. The SUA trajectories of each group were assessed, and LBW-at-term children who experienced catch-up growth had higher uric acid levels than NBW children over the entire follow-up period in the multivariable model (*p* for group effect = 0.02) (Fig. 1-b). Least squares means of each group at ages 3, 5, and 7 are presented in Table 2. Post hoc pairwise comparisons between the two groups revealed that the LBW-at-term children with catch-up growth had significantly higher uric acid levels than NBW children at ages 5 (mean difference = 0.60, *p* = 0.042) and 7 (mean difference = 0.62, *p* = 0.039).

**Table 2:** Effects of the degree of catch-up growth on serum uric acid in a mixed-model analysis

	Serum Uric Acid Level (mg/dL)					
	3 years		5 years		7 years	
Unadjusted (Mean, 95% CI)						
NBW	3.74	3.65–3.83	3.92	3.79–4.05	3.87	3.72–4.03
LBW at term & lower CUG	3.64	3.15–4.13	4.90	4.02–5.79	4.28	3.51–5.05
LBW at term & higher CUG	3.87	3.38–4.36	4.50	3.82–5.17	5.20	4.27–6.14
Adjusted^a^ (LSM, 95% CI)						
NBW	3.74	3.65–3.83	3.91	3.77–4.04	3.87	3.72–4.02
LBW at term & lower CUG	3.69	3.19–4.19	4.81	3.88–5.73	4.27	3.54–5.01
LBW at term & higher CUG	3.88	3.38–4.37	4.39	3.67–5.10	4.90	3.99–5.80

*LSM* indicates least squares means, *CI* confidence interval, *NBW* normal birth weight, *LBW* low birth weight, and *CUG* catch-up growth

^a^Results were adjusted for sex, gestational age, and body mass index at the age of uric acid measurement

To assess whether the SUA level is associated with the degree of catch-up growth, the LBW-at-term children were divided into two subgroups according to their change in weight *z*-scores from birth to age 3 using the median value. Throughout the follow-up period, the group with a weight change greater than the median value had consistently higher SUA levels than NBW children, with an increasing difference as the children aged (mean difference at age 7 = 1.03, *p* = 0.028). The unadjusted model indicated that group (*p* = 0.009), time (*p* = 0.001), and the interaction between group and time (*p* = 0.046) significantly influenced the SUA levels. The effects of group and time remained significant after adjusting for sex, gestational age, and BMI, with *p*-values of 0.037 and 0.009, respectively. The least squares means of each group at ages 3, 5, and 7 are presented in Table 2.

Based on the multivariable generalized linear model adjusted for sex, gestational age, and height, weight, and uric acid at 3 years of age, changes in the least square means of SBP between 3 and 7 years of age were higher by 7.89 mmHg with tendency towards a significant difference (*p* = 0.082) in children who experienced low birth weight and catch-up growth compared with those who had a normal birth weight. (Table 3) In addition, nearly half of children with low birth weight and catch-up growth experienced SBP changes greater than the upper limit of the 95% confidence interval for SBP change in children with normal birth weight.

**Table 3:** Effects of pre-and postnatal growth on the systolic blood pressure changes over a 7-year follow-up

	SBP at age 3			SBP at age 7			ΔSBP between age 3 to 7		
	Mean	95% CI	*p*-value	Mean	95% CI	*p*-value	Mean	95% CI	*p*-value
Unadjusted									
NBW	94.6	93.3–95.6	0.31	101.6	99.8–103.4	0.94	6.5	4.3–8.7	0.22
LBW & CUG	92.9	89.9–95.9		101.4	96.2–106.7		10.8	4.3–17.2	
Adjusted									
Model 1^a^									
NBW	94.1	92.8–95.5	0.31	101.4	99.6–103.2	0.30	6.3	4.1–8.6	0.10
LBW & CUG	95.1	91.4–98.9		105.1	98.5–111.7		13.7	5.4–22.0	
Model 2^b^									
NBW	94.1	92.7–95.4	0.58	101.2	99.0–103.3	0.95	6.0	3.7–8.3	0.08
LBW & CUG	95.3	91.5–99.1		101.6	87.8–115.5		13.9	5.7–22.1	

*SBP* indicates systolic blood pressure, *CI* confidence interval, *NBW* normal birth weight, *LBW* low birth weight; and *CUG* catch-up growth

^a^Results were adjusted for sex, gestational age, and height and weight at baseline

^b^Results were adjusted for sex, gestational age, and height, weight, and uric acid at baseline

## Discussion

This study assessed SUA levels longitudinally according to prenatal and postnatal growth characteristics. A longitudinal association was observed between LBW with accelerated postnatal growth and relatively elevated SUA levels in early childhood. Uric acid levels were consistently higher among LBW children who experienced catch-up growth compared to NBW children for the first 7 years of life. These association were statistically significant when the LBW group was limited to children who were born at term. Additionally, LBW children who experienced greater weight gain from birth to age 3 were observed to have even higher uric acid levels compared to NBW children, and the difference increased with age. Furthermore, changes in the least square means of SBP between 3 and 7 years of age were higher with tendency towards a significant difference in LBW children who experienced catch-up growth compared with those who had a normal birth weight.

Previous studies have found that LBW is an important risk factor for elevated blood pressure in children and young adults and for the development of hypertension in later life [13–15]. This study also found that the change in systolic blood pressure between age 3 and 7 years was higher among LBW children who experienced catch-up growth compared to that of NBW children, with tendency towards a significant difference. If this trend continues beyond 7 years of age, the blood pressure of LBW children can be higher than that of NBW children at later ages. To explain the association between LBW and hypertension, it has been suggested that children born with LBW often experience impaired renal development, resulting in a reduced number of nephrons at birth. Impaired nephrogenesis leads to hyperfiltration, which in turn leads to systemic hypertension [23–27]. Alternatively, vascular endothelial dysfunction [13] or altered vascular structure [28] have been proposed as the link between LBW and hypertension.

Although the mechanisms linking LBW and hypertension are not fully understood, uric acid was pointed as a possible determinant of impaired nephron development and limited glomerular endothelial cell proliferation in the previous research [29]. An increase in the uric acid level during pregnancy was associated with adverse maternal and fetal outcomes [30–32], such as preeclampsia progression and low birth weight. Bellomo et al. reported that the uric acid level was associated with preeclampsia with an OR of 8–9, and with giving birth to a small-for-gestational-age infant with an OR of 1.6–1.7 [32]. The increased uric acid in the pregnant mother passes freely into fetal circulation, and similar dietary habits and genetic factors in mother and child might increase the risk that children born with LBW would have elevated uric acid levels [1]. Our study confirmed that LBW children had continually higher SUA levels in the first 7 years of life compared to those of children born with NBW. The difference between the two groups was greater in the older age groups, even though the uric acid levels were within the range of normal for both groups.

Elevated uric acid over time may play as a significant contributor to higher blood pressure in children. In a recent cohort study, Park et al. [33] investigated the prospective association of SUA with BP in very early childhood. Children with an SUA level higher than the median values had significantly increased systolic and diastolic blood pressure at 3 years of age, and children with higher SUA levels at both 3 and 5 years of age showed higher systolic blood pressure at 7 years of age.

In addition to LBW, postnatal accelerated catch-up growth, a compensatory mechanism for intrauterine constraint, is an important factor to consider in relation to SUA and hypertension later in life. Catch-up growth is believed to be beneficial for short-term survival [34], but may cause long-term disadvantages. Postnatal accelerated growth is associated with the early development of cardiometabolic risks, including hypertension, obesity, and greater arterial wall thickness [16, 35, 36]. We observed that children who exhibited greater postnatal accelerated growth up to 3 years of age had higher uric acid levels compared to NBW children, and the difference increased with age.

The findings of this study suggest that further studies be performed to gain a deeper understanding of the association and the pathological pathways between low birth weight, uric acid, and hypertension in children, and to determine whether uric acid plays a role as a mediating factor for an early intervention of hypertension. Early intervention is critical because high blood pressure in adolescence is associated with hypertension in adulthood [6, 7].

Lifestyle changes, such as dietary modification, can help maintain an appropriate uric acid level [37–39]. Dietary zinc intake, vitamin C intake, alcohol drinking status, nutrient supplementation, and the Dietary Approaches to Stop Hypertension (DASH) diet lower SUA levels, although the effect of dietary modification on uric acid in children warrant further study.

In this study, the results were adjusted for BMI because there is evidence for a relationship between BMI and uric acid. Visceral fat accumulation, which is associated with a higher BMI, induces elevated plasma free fatty acids and stimulates triglyceride synthesis, which is followed by UA overproduction. In addition, the decrease in urinary uric acid excretion was investigated in obese or overweight people [40].

This study has several limitations that should be noted in interpreting the findings. First, because of the study design, we were unable to test the causality of the relationship between the uric acid level and hypertension but evaluated, instead, their association. Second, LBW could be because of prematurity, intrauterine growth restriction (IUGR), or both. IUGR is a dynamic concept defined by insufficient growth of the fetus in relation to its constitutional or genetically determined growth potential. Although we attempted to separate prematurity from LBW-at term, the utility of LBW was limited as a proxy measure for IUGR by the difficulty of diagnosing pathologic growth retardation of the fetus. Third, the study cohort was recruited from one university hospital, limiting the generalizability to other populations. Fourth, the relatively small sample size may limit statistical power; however, application of linear mixed models incorporated observations at all available time points and considered within-subject variation over time. Fifth, there were notably few subjects who were born with LBW but did not experience catch-up growth, and they were excluded from the study. Therefore, LBW children who experienced catch-up growth were not compared with LBW children without catch-up growth. However, this oversight may not limit our results regionally, as only approximately 10% of children born small for their gestational age fail to show catch-up growth [41]. Lastly, we could not consider subjects’ diets and mothers’ medical histories.

## Conclusions

The prospective cohort design allowed individuals to be tracked longitudinally to monitor the SUA level and blood pressure from birth to age 7. Follow-up of subjects up to age 7 is an improvement over previous cross-sectional studies. Children born with LBW who experienced a catch-up growth had increased uric acid and higher changes in SBP than did NBW children, with tendency towards a significant difference. Further research is needed to explore whether early intervention for these children to maintain appropriate SUA levels reduces the risk of developing hypertension later in life.

## Supplementary information

**Additional file 1: Figure S1.** Q-Q plot for normality test.

**Additional file 2: Figure S2.** Plots of the residuals versus variables for linearity test.

## Data Availability

The datasets used and/or analysed during the current study are available from the corresponding author on reasonable request.

## Abbreviations

BMI: Body mass index
CUG: Catch-up growth
DASH: Dietary Approaches to Stop Hypertension
LBW: Low birth weight
NBW: Normal birth weight
SBP: Systolic blood pressure
SUA: Serum uric acid
