# Supplementary material for: Association between pre-and postnatal growth and longitudinal trends in serum uric acid levels and blood pressure in children aged 3 to 7 years
Source: BMC Pediatr. 2020 Jan 20;20:23. doi: 10.1186/s12887-020-1922-8 (PMC6971928; doi:10.1186/s12887-020-1922-8)
Supplement: Supplementary file 1 — Additional file 1: Figure S1. Q-Q plot for normality test. [file 12887_2020_1922_MOESM1_ESM.pptx]

## Slide 1
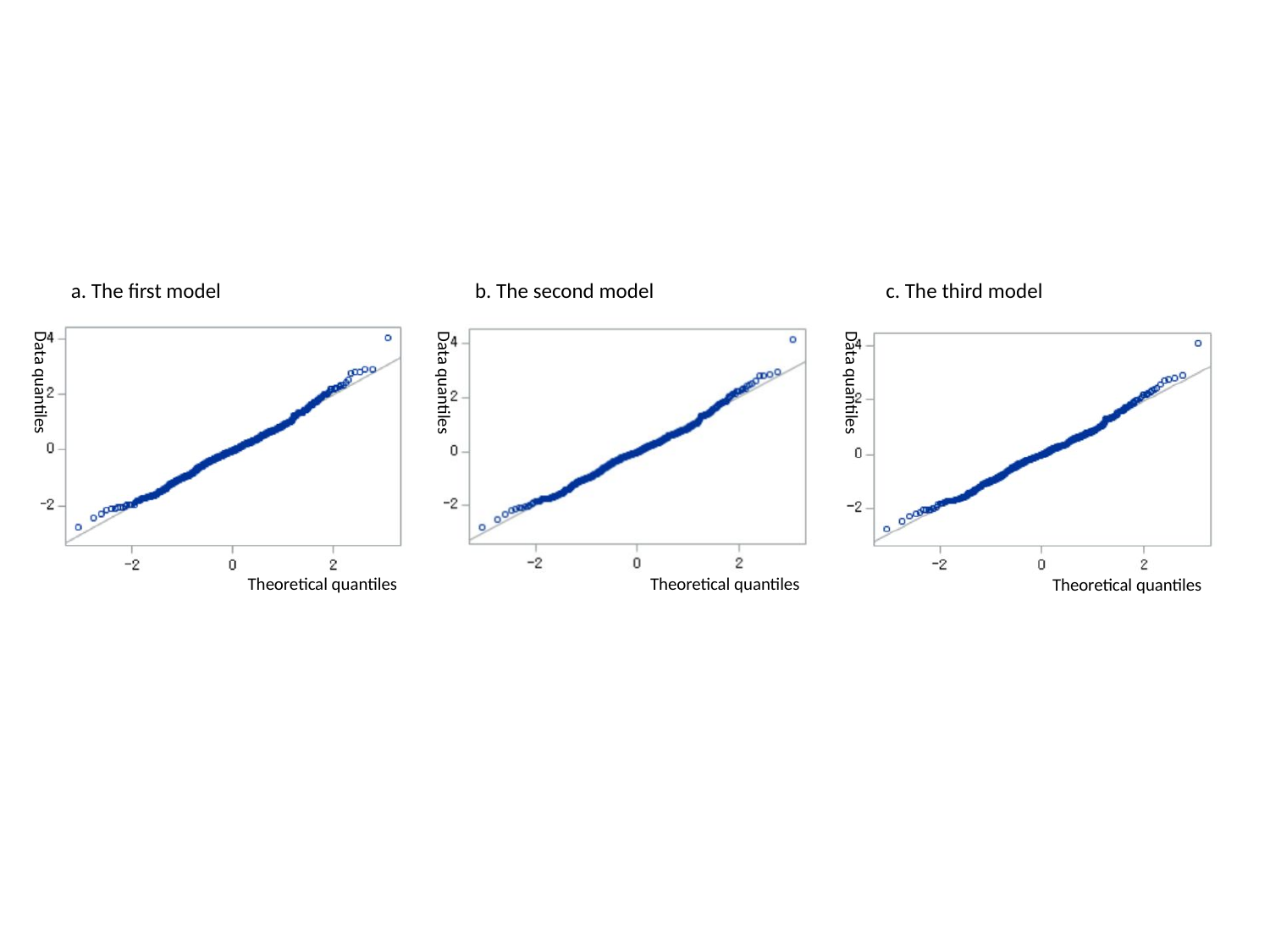

a. The first model
b. The second model
c. The third model
Data quantiles
Data quantiles
Data quantiles
Theoretical quantiles
Theoretical quantiles
Theoretical quantiles
