# Supplementary material for: Association between pre-and postnatal growth and longitudinal trends in serum uric acid levels and blood pressure in children aged 3 to 7 years
Source: BMC Pediatr. 2020 Jan 20;20:23. doi: 10.1186/s12887-020-1922-8 (PMC6971928; doi:10.1186/s12887-020-1922-8)
Supplement: Supplementary file 2 — Additional file 2: Figure S2. Plots of the residuals versus variables for linearity test. [file 12887_2020_1922_MOESM2_ESM.pptx]

## Slide 1
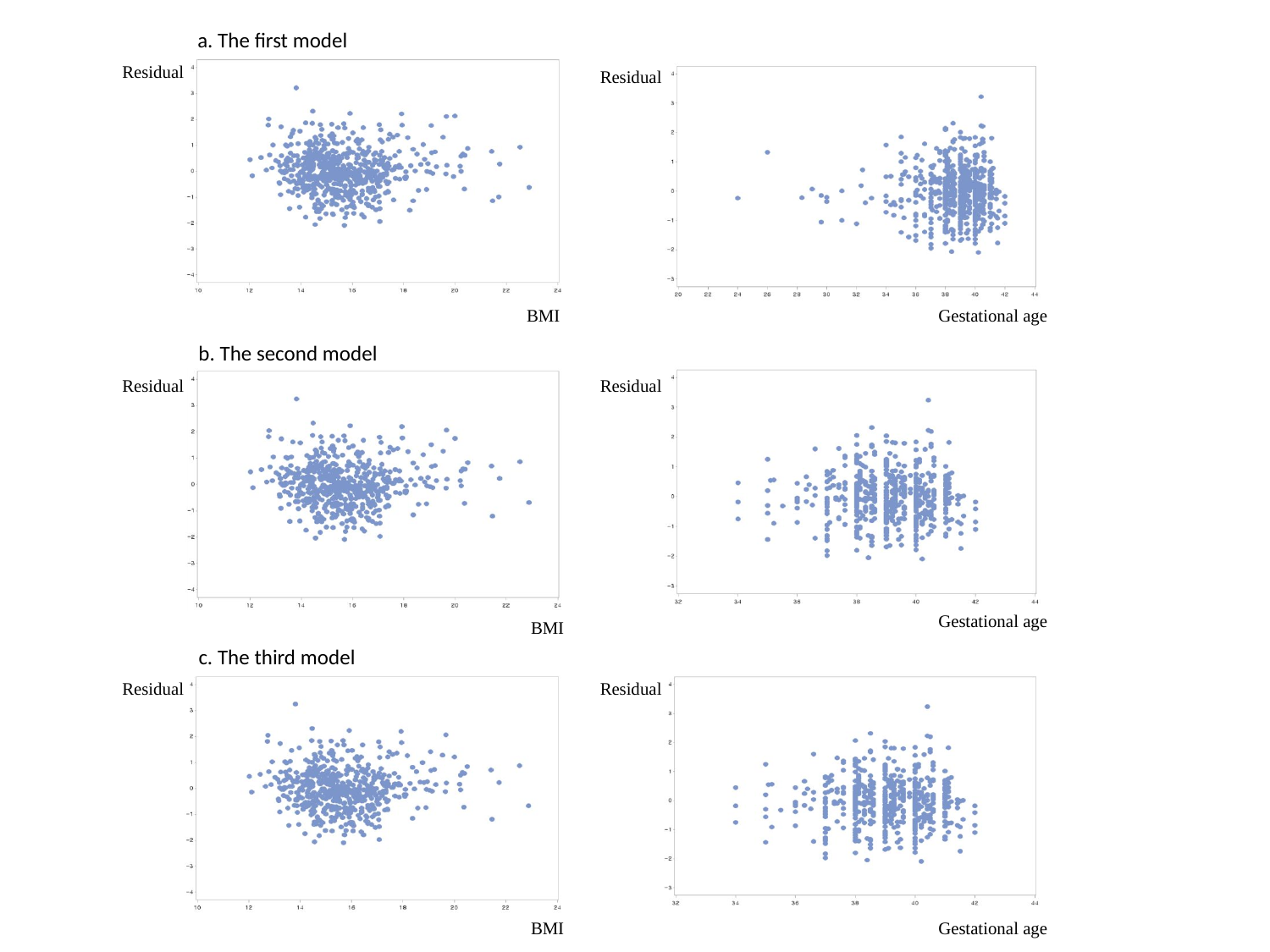

a. The first model
Residual
Residual
BMI
Gestational age
b. The second model
Residual
Residual
Gestational age
BMI
c. The third model
Residual
Residual
BMI
Gestational age
